# Postoperative Venous Thromboembolism Prophylaxis by General Surgeons in a Developing Country: A Survey

**DOI:** 10.1155/2013/873750

**Published:** 2013-09-19

**Authors:** Aniketh Venkataram, Shivashankar Santhosh, Deevish Dinakar, Shivananda Siddappa, Rajashekara Babu, Sadashivaiah Shivaswamy

**Affiliations:** Department of General Surgery, Bangalore Medical College and Research Institute, No. 182, 100 Feet Ring Road, Banashankari 3rd Stage, Bangalore 560085, India

## Abstract

Venous thromboembolism (VTE) is the most common preventable cause of hospital death. Several audits in the west have demonstrated that appropriate thromboprophylaxis is not being offered to a large number of surgical patients. Similar audits are lacking in the Indian population, and a perception exists among Indian surgeons that Indian patients are not susceptible to VTE. Hence we undertook a survey to analyze the existing knowledge and practice of VTE prophylaxis amongst general surgeons in India. A questionnaire-based survey was conducted on 100 active general surgeons. We found that 97% of surgeons had encountered VTE in their practice, and 49% had encountered mortality from pulmonary embolism. 64% of surgeons do not routinely score patients preoperatively for their VTE risk, and only 33% reported the presence of an institute-based protocol for the same. There was a wide disparity in the prophylaxis methods used for each risk group, particularly in the moderate-risk group. These findings suggest the need for adoption of institute-based protocols for prophylaxis and the evolution of Indian guidelines for VTE prophylaxis.

## 1. Introduction

Venous thromboembolism (VTE), including deep venous thrombosis (DVT) and pulmonary embolism (PE), is an important preventable cause for morbidity and mortality among surgical patients. Several studies have identified PE as the most common preventable cause of hospital death [1–3]. The prevention of VTE is the number one strategy to improve patient care according to the Agency for Health Care Research and Quality [4].

 Due to the often clinically silent nature of VTE and its increased risk and potential morbidity and mortality in surgical patients, routine thromboprophylaxis is advocated in western countries. The most commonly followed protocols are those established by the American College of Chest Physicians (ACCP) which were recently revised in 2012 [5]. In spite of the establishment of these protocols and availability of a number of safe prophylactic agents, numerous audits have shown that adequate prophylaxis is not being offered to a large number of surgical patients [6–8].

 Traditionally, VTE has been thought to be less common in Asian populations, particularly in Indians. Few small, uncontrolled studies largely performed on orthopaedic patients supported this belief [9, 10]. As a result, there were little awareness or practice of VTE prophylaxis in India and a lack of established guidelines for Indian patients. However recent studies reveal that the incidence of VTE in Indian patients is comparable with that in the rest of the world [11]. There has been very little published data on the prophylaxis methods being adopted in India, and it is unclear whether there is any consensus on the protocols being followed for prophylaxis in the country.

 Hence we undertook our survey to determine the experience of Indian surgeons with VTE, their awareness regarding VTE prophylaxis, and their protocols for the same.

## 2. Materials and Methods

 A questionnaire was created in consultation with general surgeons, vascular surgeons, and pulmonologists (Supplementary Figure 1 available online at http://dx.doi.org/10.1155/2013/873750). This covered various aspects including

- surgeons prior experience with DVT and PE;
- surgeons experience with mortality from VTE;
- diagnostic methods used for VTE;
- surgeons beliefs regarding incidence of VTE in Indian populations and need for prophylaxis;
- concerns regarding prophylaxis;
- presence of institute-based protocols;
- prophylaxis methods used for each risk group.

This questionnaire was distributed to a cross section of practicing consultant general surgeons in the city of Bangalore, south India, at the monthly surgical society of Bangalore meetings. This ensured coverage of a cross section of surgeons from all hospitals including both the public and private sectors. Various surgical specialities like orthopaedic, plastic, neuro, and cardiac surgeons were excluded from this study owing to the differences in prophylaxis protocols advised for each of these groups.

 A total of 100 questionnaires were distributed, collected, and analyzed using descriptive statistics.

## 3. Results

The results of the survey were as follows.

### 3.1. VTE Incidence (Figure 1)

An overwhelming 93% of surgeons reported that they had encountered VTE in their clinical practice, with only 7% not having seen any episodes of VTE. 50% of surgeons said they had seen VTE in less than 1% of their cases, 38% of surgeons in 1–5% of their cases, and 5% reported they had encountered VTE in more than 5% of their cases. 

 55% of surgeons believed that Indians were at a lower risk than Caucasians for VTE. Interestingly, 37% of surgeons felt that Indians were at the same risk as Caucasians for VTE with 8% saying they were not sure.

 All surgeons relied on clinical symptoms and venous Doppler to diagnose VTE postoperatively. 38% said they used D-dimer assay, while only 9% used scoring criteria.

### 3.2. Pulmonary Embolism (Figure 1)

73% of surgeons had encountered PE, and 49% of surgeons had encountered mortality from PE in their clinical practice.

### 3.3. Prophylaxis Protocols (Figure 2)

Only 36% of surgeons said that they routinely scored patients preoperatively on their VTE risk, with 64% of all surgeons not scoring patients. Only 33% of surgeons reported that there was an institute-based protocol for VTE prophylaxis at their centre. Of the remaining 67% (who did not have an institute-based protocol) 97% believed that the same should be in place. The most common concerns expressed regarding VTE prophylaxis were burden of cost (44%) and risk of postoperative bleeding (24%).

### 3.4. Prophylaxis Methods Used (Figure 3)

For low-risk patients, 83% said they would give no specific prophylaxis other than early ambulation, and 11% said they advised mechanical prophylaxis with either compression stockings or intermittent compression devices. 7% said they would advise low-molecular-weight heparin (LMWH).

 There was a wide disparity in the prophylaxis methods used for moderate-risk patients. 34% of surgeons advised no specific prophylaxis other than early ambulation. 19% advised mechanical prophylaxis preferably with intermittent compression devices, and 13% advised pharmacological prophylaxis (preferably with LMWH). Another 34% advised a combination of mechanical and pharmacological prophylaxis.

 For high-risk patients, 77% of surgeons said they would advise a combination of mechanical and pharmacological prophylaxis. 19% advised only LMWH, and 4% only mechanical prophylaxis.

## 4. Discussion

### 4.1. VTE Incidence

The incidence of VTE has traditionally been considered to be lower in Asian populations as compared to western ones. However recent studies have challenged this perception with increasing incidence of VTE being reported. One reason for this being that earlier Asian studies tended to report only those VTE events that were symptomatic. Whereas western studies documented both symptomatic as well as asymptomatic events on the basis of bilateral venography, leading to the impression of lower risk among Asian populations.

Leizorovicz et al. were the first to suggest that VTE was a significant problem in Asians [11]. This paved the way for numerous other prospective studies in Asian countries which reported rising rates of VTE and PE with incidences around 17–20/1000 [12, 13]. The SMART study, which covered 2420 Asian patients undergoing major orthopaedic surgery without prophylaxis, revealed an incidence of symptomatic VTE of 2.3% which is comparable with western populations [14]. These later studies documented the incidence of asymptomatic VTE using venography. Asymptomatic DVT, though considered less important than symptomatic VTE, can severely compromise the quality of life of patients years after the event. 

 In our survey, 93% of surgeons had encountered VTE, and 49% had seen mortality from PE. This goes against the previously held belief that Indian patients are not susceptible to VTE. Indeed, 37% of the respondents in our survey believed that Indians were at the same risk for VTE as western populations.

 Indian studies on the subject are largely inadequate and conflicting, with older studies showing lower rates of VTE incidence [9, 10] and newer ones higher. A recent study conducted at CMC Vellore reported an incidence of 17.46/10000 hospital admissions which is comparable to other Asian results [15]. This suggests that the perceived lower incidence in Indians might in fact be due to a lack of awareness and inadequate diagnostic facilities. An autopsy study performed on 1000 patients at PGI Chandigarh in north India found fatal pulmonary embolism in 16% of cases [16]. In the Indian subset data of the global Prospective Registry On Venous thromboembolic Events (PROVE) registry, VTE events were found to occur as frequently as in the rest of the world [17].

 The findings of our survey suggest that routine thromboprophylaxis for all cases in Indians should be considered. Epidemiological studies to determine the incidence of VTE in Indians are also warranted.

### 4.2. Diagnosis of VTE

All surgeons relied on clinical symptoms and venous Doppler to diagnose VTE postoperatively. 38% said they used D-dimer assay. The ACCP recommends that the diagnostic test is dependent on the pretest probability of VTE. In low-to-moderate VTE risk, D-dimer is advocated as the diagnostic test of choice. In high VTE risk whole-leg ultrasound is preferred [18].

### 4.3. Prophylaxis Protocols

64% of surgeons said that they do not score patients preoperatively for VTE, and 67% responded that there was no institute-based protocol for VTE prophylaxis at their center. 97% of these surgeons believed that an institute-based protocol should be in place.

 The ACCP advocates that each institute adopt its own protocol for VTE prophylaxis [5]. Despite the establishment of guidelines and protocols, numerous studies have shown that adequate prophylaxis is not being offered to a large number of surgical patients across the world. The most significant of these is the ENDORSE study which included 30827 patients from 32 countries including India. Out of 19842 surgical patients at risk for VTE, 41.5% of cases did not receive proper prophylaxis [6]. In the Indian patients enrolled in the ENDORSE study, only 16.3% of at-risk surgical patients received adequate prophylaxis [19]. 

44% of the respondents in our study said they believed thromboprophylaxis would add to the burden of cost, and 24% were afraid of postoperative bleeding. Another study performed on intensive care medical and surgical patients in Mumbai found that only 47% of ICU patients received pharmacological prophylaxis for fear of bleeding [20].

### 4.4. Prophylaxis Methods

Our study revealed inadequate knowledge among surgeons regarding the prophylaxis methods used for each risk group. There was a wide disparity in the methods used particularly in the moderate-risk group patients, where 34% of surgeons said they would give no prophylaxis, and an equal 34% said they would advise a combination of mechanical and pharmacological prophylaxis. 

 These findings suggest that efforts need to be taken to improve the awareness among surgeons regarding how to score patients for their VTE risk and regarding the appropriate prophylaxis methods to be used for each risk group. The disparity in methods used might also in part be attributed to a lack of national guidelines for VTE prophylaxis. 

 In 2010, the Asia-Pacific Thrombosis Advisory Board released a consensus paper which recognized that VTE is an important issue in Asian patients and advised that all Asian countries adopt their own national guidelines for prophylaxis or, in the absence of the same, adopt the ACCP guidelines [21]. Several Asian countries including Japan and Korea have adopted their own national guidelines for VTE prophylaxis [22, 23]. These follow the ACCP guidelines for prophylaxis but differ in the fact that they score their populations at one level higher than those recommended by the ACCP.

In view of the evidence documenting the risk for VTE in Indians, evolution of Indian guidelines for VTE prophylaxis appears to be in order. Many surgeons believe that the same scoring criteria used for western populations would not be appropriate for Indian patients and are hence reluctant to use ACCP guidelines. Adoption of our own national guidelines would increase awareness for VTE and ensure uniformity in prophylaxis protocols used.

Our survey was limited by the fact that it was conducted in only one city in south India. At present we are in the process of expanding the survey to cover other cities. Moving forward, we believe there is a need to increase awareness among general surgeons regarding the need for VTE prophylaxis and the adoption of institute-based protocols for the same. At a national level, epidemiological studies should be undertaken to ascertain the risk of Indian populations for VTE, and national guidelines for prophylaxis need to be established.

## 5. Conclusions

Our survey was aimed at analyzing the knowledge, attitude, and practices of Indian surgeons for VTE prophylaxis. We found that almost all surgeons had encountered VTE, and nearly half had seen mortality from the same. We found that most surgeons do not score patients preoperatively for their risk and most centres do not have institute-based protocols. We also found inadequate knowledge among surgeons regarding prophylaxis methods and a lack of uniformity in the currently used methods. We would advocate for greater awareness and education of surgeons on this topic and the adoption of national guidelines for VTE prophylaxis. With these steps, this major cause of morbidity and mortality in surgical patients can easily be prevented.

## Supplementary Material

POSTOPERATIVE VENOUS THROMBOEMBOLISM PROPHYLAXIS SURVEY QUESTIONNAIRE

## Figures and Tables

**Figure 1:**
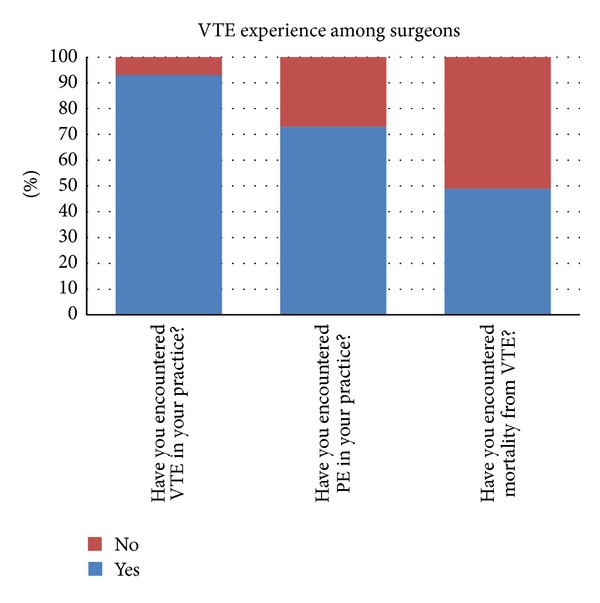
VTE experience among surgeons.

**Figure 2:**
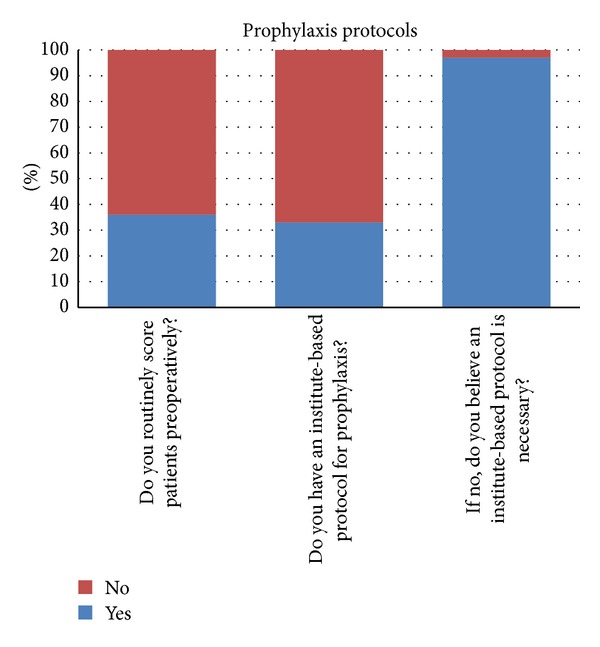
Prophylaxis protocols.

**Figure 3:**
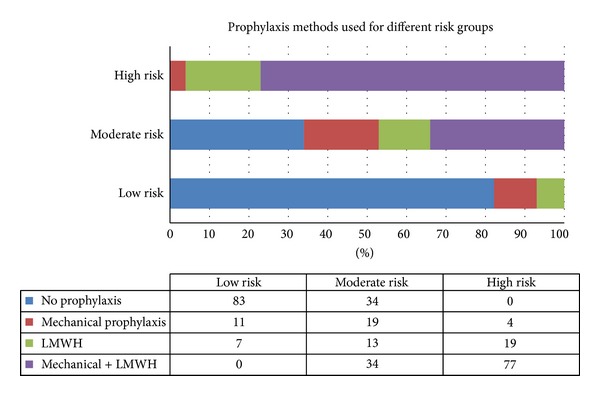
Prophylaxis methods used for different risk groups.

## References

[1] Lindblad B, Eriksson A, Bergqvist D (1991). Autopsy-verified pulmonary embolism in a surgical department: analysis of the period from 1951 to 1988. *British Journal of Surgery*.

[2] Stein PD, Henry JW (1995). Prevalence of acute pulmonary embolism among patients in a general hospital and at autopsy. *Chest*.

[3] White RH, Zhou H, Romano PS (2003). Incidence of symptomatic venous thromboembolism after different elective or urgent surgical procedures. *Thrombosis and Haemostasis*.

[4] Shojania KG, Duncan BW, McDonald KM (2012). Making health care safer: a critical analysis of patient safety practices. *Report/Technology Assessment*.

[5] Gould MK, David A, Wren SM (2012). Prevention of VTE in nonorthopedic surgical patients: antithrombotic therapy and preventionof thrombosis, 9th ed, American College of Chest PhysiciansEvidence-Based Clinical Practice Guidelines. *Chest*.

[6] Cohen AT, Tapson VF, Bergmann J-F (2008). Venous thromboembolism risk and prophylaxis in the acute hospital care setting (ENDORSE study): a multinational cross-sectional study. *The Lancet*.

[7] Kahn SR, Panju A, Geerts W (2007). Multicenter evaluation of the use of venous thromboembolism prophylaxis in acutely ill medical patients in Canada. *Thrombosis Research*.

[8] Tapson VF, Hyers TM, Waldo AL (2005). Antithrombotic therapy practices in US hospitals in an era of practice guidelines. *Archives of Internal Medicine*.

[9] Jain V, Dhaon DK, Jaiswal A, Nigam V, Singla J (2004). Deep vein thrombosis after total hip and knee arthroplasty in Indian patients. *Postgraduate Medical Journal*.

[10] Bagaria V, Modi N, Panghate A, Vaidya S (2006). Incidence and risk factors for development of venous thromboembolism in Indian patients undergoing major orthopaedic surgery: results of a prospective study. *Postgraduate Medical Journal*.

[11] Leizorovicz A, Turpie AGG, Cohen AT, Dhillon KS, Angchaisuksiri P, Wang C-J (2004). Epidemiology of post-operative venous thromboembolism in Asian countries. *International Journal of Angiology*.

[12] Lee LH, Gu KQ, Heng D (2002). Deep vein thrombosis is not rare in Asi: the Singapore General Hospital experience. *Annals Academy of Medicine Singapore*.

[13] Piovella F, Wang C-J, Lu H (2005). Deep-vein thrombosis rates after major orthopedic surgery in Asia. An epidemiological study based on postoperative screening with centrally adjudicated bilateral venography. *Journal of Thrombosis and Haemostasis*.

[14] Leizorovicz A, Turpie AGG, Cohen AT, Wong L, Yoo MC, Dans A (2005). Epidemiology of venous thromboembolism in Asian patients undergoing major orthopedic surgery without thromboprophylaxis. The SMART Study. *Journal of Thrombosis and Haemostasis*.

[15] Lee AD, Stephen E, Agarwal S, Premkumar P (2009). Venous Thrombo-embolism in India. *European Journal of Vascular and Endovascular Surgery*.

[16] Kakkar N, Vasishta RK (2008). Pulmonary embolism in medical patients: an autopsy-based study. *Clinical and Applied Thrombosis/Hemostasis*.

[17] Pinjala RK, Agarwal MB (2005). Turpie AGG; for PROVE investigators. A characterization of patients with symptomatic DVT in India. *Journal of Thrombosis and Haemostasis*.

[18] Bates SM, Jaeschke R, Stevens SM (2012). Diagnosis of DVT: antithrombotic therapy and prevention of thrombosis, 9th ed: American College of Chest Physicians evidence-based clinical practice guidelines. *Chest*.

[19] Pinjala R, Agnihotri V, Balraj A (2012). Venous thromboembolism risk & prophylaxis in the acute hospital care setting (ENDORSE), a multinational cross-sectional study: results from the Indian subset data. *Indian Journal of Medical Research*.

[20] Ansari K, Dalal K, Patel M (2007). Risk stratification and utilisation of thrombo-embolism prophylaxis in a medical-surgical ICU: a hospital-based study. *Journal of the Indian Medical Association*.

[21] Cohen AT (2010). Asia-Pacific Thrombosis Advisory Board consensus paper on prevention of venous thromboembolism after major orthopaedic surgery. *Thrombosis and Haemostasis*.

[22] Bang SM, Jang MJ, Oh D (2010). Korean Society of Thrombosis and Hemostasis. Korean guidelines for the prevention of venous thromboembolism. *Journal of Korean Medical Science*.

[23] The Korea Society on Thrombosis and Hemostasis (2009). Japanese guideline for prevention of venous thromboembolism. *Korean Journal of Thrombosis and Hemostasis*.

