# Supplementary material for: Postoperative Venous Thromboembolism Prophylaxis by General Surgeons in a Developing Country: A Survey
Source: Thrombosis. 2013 Sep 19;2013:873750. doi: 10.1155/2013/873750 (PMC3818923; doi:10.1155/2013/873750)
Supplement: Supplementary file 1 — POSTOPERATIVE VENOUS THROMBOEMBOLISM PROPHYLAXIS SURVEY QUESTIONNAIRE [file 873750.f1.pdf]

POSTOPERATIVE VENOUS THROMBOEMBOLISM PROPHYLAXIS SURVEY QUESTIONNAIRE

Respected Sir/ Madam

Venous thromboembolism (VTE) including deep venous thrombosis(DVT) and pulmonary embolism(PE) is reported to be the most common cause of preventable hospital death. Evidence advocates the use of prophylaxis for at risk patients, thereby reducing morbidity and mortality. For this reason we, from the department of General Surgery, Bangalore medical college, are conducting a survey regarding VTE prophylaxis knowledge and practices among surgical specialities in Bangalore, to analyze and comment upon the existing methods of VTE prophylaxis in Bangalore. We would be grateful if you could fill in the following questionnaire regarding the practice at your center.

Name- ____

E mail- ____

Designation- ____

Institute- ____

1) Have you encountered cases of postoperative DVT in your surgical practice?
   a. Yes □ b. No □

2) If yes, in what percentage of surgical cases have you encountered DVT?
   a. <1% □ b.1-5% □ c.5-10% □ d.>10% □

3) How do you diagnose postoperative DVT (may mark more than one)?
   a. Clinical symptoms □
   b. Scoring criteria □
   c. D-dimer assay □
   d. Venous Doppler □
   e. Venography □
   f. Other (please specify) ______________________________ □

4) Have any of your patients had postoperative pulmonary embolism?
   a. Yes □ b. No □

5) If yes, have you encountered any mortality from pulmonary embolism?
   a. Yes □ b. No □

6) In your experience, how prone are Indians to VTE?
   a. Same as western populations □
   b. Less than western populations □

7) Do you routinely give VTE prophylaxis for 'at risk' surgical patients?
   a. Yes □ b. No □

8) If no, for what reason?
   a. No requirement in Indian patients due to lower risk □
   b. Risk of postoperative haemorrhage □
   c. Burden of cost □
   d. Difficulty in monitoring □
   e. Other (please specify) ______________________________ □

9) Do you routinely score patients preoperatively on their VTE risk?
   a. Yes □ b. No □

10) If center based, is there an institute based protocol for VTE prophylaxis at your center?
   a. Yes □ b. No □

11) If no, do you believe there should be an institute based protocol?
   a. Yes □ b. No □

12) In which of the following categories do you give prophylaxis and what method do you use? (May mark more than one)

Low risk □	Moderate risk □	High Risk □
□ None<br>□ Early ambulation<br>□ Compression stockings<br>□ Intermittent compression devices<br>□ Low molecular weight heparin (LMWH)<br>□ Warfarin<br>□ Other (please specify)<br>____________________	□ None<br>□ Early ambulation<br>□ Compression stockings<br>□ Intermittent compression devices<br>□ LMWH<br>□ Unfractionated heparin<br>□ Warfarin<br>□ Rivaroxaban<br>□ Other ____________________	□ None<br>□ Early ambulation<br>□ Compression stockings<br>□ Intermittent compression devices<br>□ LMWH<br>□ Unfractionated heparin<br>□ Warfarin<br>□ Rivaroxaban<br>□ Other ____________________

Signature
